# Physical Activity Reduces Epilepsy Incidence: a Retrospective Cohort Study in Swedish Cross-Country Skiers and an Experimental Study in Seizure-Prone Synapsin II Knockout Mice

**DOI:** 10.1186/s40798-019-0226-8

**Published:** 2019-12-16

**Authors:** Matilda Ahl, Una Avdic, Maria Compagno Strandberg, Deepti Chugh, Emelie Andersson, Ulf Hållmarker, Stefan James, Tomas Deierborg, Christine T. Ekdahl

**Affiliations:** 1 Inflammation and Stem Cell Therapy Group, Division of Clinical Neurophysiology, Lund University, BMC A11, Sölvegatan 17, SE-221 84 Lund, Sweden; 2 Lund Epilepsy Center, Department of Clinical Science, Lund University, Lund, Sweden; 3 Experimental Neuroinflammation Laboratory, Department of Experimental Medical Science, Lund University, Lund, Sweden; 4 Department of Medical Sciences, Cardiology, Uppsala University, Uppsala, Sweden; 5 Department of Internal Medicine, Mora Hospital, Mora, Sweden

**Keywords:** Exercise, Physical activity, Epilepsy incidence, Seizures, Epileptogenesis, Epilepsy

## Abstract

**Background:**

Epilepsy patients commonly exercise less than the general population. Animal studies indicate beneficial effects of physical activity in established epilepsy, while its effect on the development is currently less known.

**Methods:**

Here, we investigated the incidence of epilepsy during 20 years in a cohort of participants from the long-distance Swedish cross-country ski race Vasaloppet (*n* = 197,685) and compared it to the incidence of non-participating-matched controls included in the Swedish population register (*n* = 197,684). Individuals diagnosed with diseases such as stroke and epilepsy before entering the race were excluded from both groups. Experimentally, we also determined how physical activity could affect the development of epilepsy in epilepsy-prone synapsin II knockout mice (SynIIKO), with and without free access to a running wheel.

**Results:**

We identified up to 40–50% lower incidence of epilepsy in the Vasaloppet participants of all ages before retirement. A lower incidence of epilepsy in Vasaloppet participants was seen regardless of gender, education and occupation level compared to controls. The participants included both elite and recreational skiers, and in a previous survey, they have reported a higher exercise rate than the general Swedish population. Sub-analyses revealed a significantly lower incidence of epilepsy in participants with a faster compared to slower finishing time. Dividing participants according to specified epilepsy diagnoses revealed 40–50% decrease in focal and unspecified epilepsy, respectively, but no differences in generalized epilepsy. Voluntary exercise in seizure-prone SynIIKO mice for 1 month before predicted epilepsy development decreased seizure manifestation from > 70 to 40%. Brain tissue analyses following 1 month of exercise showed increased hippocampal neurogenesis (DCX-positive cells), while microglial (Iba1) and astrocytic activation (GFAP), neuronal Map2, brain-derived neurotrophic factor and its receptor tyrosine receptor kinase B intensity were unaltered. Continued exercise for additionally 2 months after predicted seizure onset in SynIIKO mice resulted in a 5-fold reduction in seizure manifestation (from 90 to 20%), while 2 months of exercise initiated at the time of predicted seizure development gave no seizure relief, suggesting exercise-induced anti-epileptogenic rather than anti-convulsive effect.

**Conclusion:**

The clinical study and the experimental findings in mice indicate that physical activity may prevent or delay the development of epilepsy.

## Key points

- In a large retrospective cohort of participants in the Swedish cross-country ski race Vasaloppet (*n* = 197,685), we identified a 50% lower incidence of epilepsy in skiers at all ages, regardless of gender, education and occupation level compared to non-participating matched individuals from the general Swedish population (*n* = 197,684).
- In epilepsy-prone synapsin II knockout mice, we observed a > 50% reduction in numbers of epileptic seizures and delayed epilepsy development following voluntary exercise.

## Background

Epilepsy is a heterogeneous neurological disease affecting almost 1% of the population worldwide. It is characterized by unpredicted recurrent spontaneous seizures and associated with an increased risk of injuries, cognitive deficits, mood disorders and mortality. Treatment is symptomatic and the plethora of aetiologies varies from acquired brain trauma/stroke/tumours/infections to genetic predisposition, neurodevelopmental/degenerative and metabolic disorders. People with epilepsy commonly exercise less than the general population due to fear of inducing seizures and fear of physical injury [1, 2]. However, besides the overall health benefits related to exercise, it has also been suggested to be favourable for established seizure disorders [3–5]. Robust clinical and experimental studies on whether the development of epilepsy (epileptogenesis; e.g. time window before first spontaneous seizure) is affected by physical activity are few.

Exercise-related physiological mechanisms affecting the brain are just beginning to be revealed. High intensity and chronic exercise induce inflammatory changes and altered neuroendocrine responses within the hypothalamic-pituitary-adrenal (HPA) hormonal axis in humans [6–8]. Physically active rodents also show an increased HPA-axis drive, changes in blood levels of corticosterone and neurotrophic factors [9–15], and altered brain pathology [16] including anti-inflammatory features in the brain [17, 18]. Animal models of epilepsy, i.e. the pilocarpine model, have shown exercise-associated beneficial effects such as decreased seizure frequency [19, 20]. In both the kainic acid, pilocarpine and electrical kindling model of epilepsy, exercise may also increase seizure threshold [21–23] and in a genetic model of absence epilepsy, the WAG/Rij rats, swimming exercise decreased the amount of epileptiform activity in electroencephalographic recordings [24]. The results indicate primarily an anti-convulsive effect of exercise, since the animals already exhibited seizures upon inclusion. However, there are a few studies on chemically induced seizures investigating the epileptogenic phase following exercise [25–27]. These studies present delayed latency to seizures and less severe motor symptoms, which indicates that exercise may interfere also with disease development.

In this study, we investigated the incidence of epilepsy in a uniquely large cohort of physically active participants in a Swedish long-distance ski race (Vasaloppet) and compared it to the incidence in a non-participating matched control group. In the same cohort of participants (skiers) and non-participants (controls), we have previously shown lower risk of death of all causes and decreased recurrent myocardial infarction, while occurrence of recurrent stroke remained unaltered [28–30]. Vasaloppet is a physically challenging race, including annually over 50,000 elite and recreational skiers. To delineate our cohort, patients with diagnosed epilepsy or severe diseases such as stroke and chronic neurological diseases before entering the race (skiers) or at the time of inclusion (controls) were excluded from the study.

To further investigate the timing of initiation of physical activity and epileptogenesis, we also studied epilepsy-prone genetically modified mice lacking synapsin II (SynIIKO), a model of focal epilepsy with secondary generalization. Mutations in the synapsin family have been found in patients with epilepsy [31, 32]. SynIIKO mice exhibit age-dependent development of epileptic seizures in stressful situations such as human handling. The SynIIKO mice were provided with running wheels for voluntary physical exercise at different time points during both epilepsy development and progression. Time to seizure onset, seizure frequency and severity were analysed along with neurotrophic factor expression, neuronal and glial reactions in brain tissue and corticosterone levels in faeces.

## Methods

### Study Design and Participating Subjects in Cohort Study

From year 1989–2010, 126,362 males (62%) and 77,447 females (38%) participated in at least one Vasaloppet race. The majority (55%) of skiers participated in the 90-km race and the rest in 30/45-km races. Data collected from Vasaloppet register included name, Swedish personal identification number and finishing time. As a control group, men and women were randomly selected from the general population register of Sweden (non-participating controls) and frequency-matched to the participating skiers according to age (5-year intervals), gender, region and year of race. Individuals with a severe disease such as stroke and chronic neurological disease were excluded from the study [30], and the estimated survival, Charlson’s index, of both skiers and controls was calculated (Table 1). This left a cohort of 197,684 controls and 197,685 participating skiers. For information regarding the demographics, see Table 1. A digital survey [33] among the participants of the ski race in 2006 (*n* = 5180 women and 7061 men; 62% of participants) compared to age-matched answers (*n* = 21,444 women and 18,558 men) in the annual survey for the Swedish Health register in 2006–2007 showed differences in both life style and exercise habits between the groups. In the skiing participating group, 56–65% (men-women) exercised > 4 h a week, which was significantly higher than 17–18% (men-women) exercising > 1.5 h a week reported in the Swedish Health register. Life style factors such as smoking was lower among the participating skiers (smoking daily, skiers 1–2% (men-women) vs the Swedish Health register 13–15% (men-women)) and intake of fruits and vegetables > 5 times a week was higher (12–33% men-women) compared to the Swedish Health register (6–14% men-women).

**Table 1:** Overview of participating skiers and non-participating controls. Individuals in the dataset divided into gender, age group, year of inclusion, family status, education level, occupational level and Charlson index. Charlson index reflects estimated survival rate in each group [45]

Variable	*N*	Controls	Skiers	Total	*P* value
		*N* = 197,684	*N* = 197,685	*N* = 395,369	
Gender					
Woman	395,369	74,899 (37.9%)	74,897 (37.9%)	149,796 (37.9%)	0.991^1^
Age	395,369	36.0 (29.0–46.0)	36.0 (29.0–46.0)	36.0 (29.0–46.0)	0.362^2^
Age group:					
20–30	395,369	63,238 (32.0%)	63,238 (32.0%)	126,476 (32.0%)	1.00^1^
30–40		58,246 (29.5%)	58,246 (29.5%)	116,492 (29.5%)	
40–50		45,957 (23.2%)	45,958 (23.2%)	91,915(23.2%)	
50–100		30,243 (15.3%)	30,243 (15.3%)	60,486 (15.3%)	
Year	395,369	2001 (1996–2006)	2001 (1996–2006)	2001 (1996–2006)	1.00^2^
Year group:					
1991–2000	384,601	89,517 (46.6%)	89,518 (46.6%)	179,035 (46.6%)	
2001–2005		51,752 (26.9%)	51,752 (26.9%)	103,504 (26.9%)	
2006–2010		51,031 (26.5%)	51,031 (26.5%)	102,062 (26.5%)	
Family status:					
Cohabiter	384,573	108,629 (56.5%)	115,757 (60.2%)	224,386 (58,3%)	< 0.001^1^*
Educational level					
Primary school	392,048	34,806 (17.9%)	14,538 (7.4%)	49,344 (12.6%)	< 0.001^1^*
High school		99,936 (51.3%)	76,635 (38.8%)	176,571 (45.0%)	
University		59,986 (30.8%)	106,147 (53.8%)	166,133 (42.4%)	
Occupation level					
Employed	378,596	142,020 (75.5%)	162,849 (85.5%)	304,869 (80.5%)	< 0.001^1^*
Unemployed		43,811 (23.3%)	25,662 (13.5%)	69,473 (18.4%)	
Retired		2225 (1.2%)	2029 (1.1%)	4254 (1.1%)	
Charlson index					
0	2,407,956	112,182 (91.7%)	111,061 (93.8%)	223,243 (92.7%)	< 0.001^1^*
1		9030 (7.4%)	7018 (5.9%)	16,048 (6.7%)	
2		839 (0.7%)	322 (0.3%)	1161 (0.5%)	
3		197 (0.2%)	53 (0.0%)	250 (0.1%)	
4		43 (0.0%)	4 (0.0%)	47 (0.0%)	
5		19 (0.0%)	2 (0.0%)	21 (0.0%)	
6		17 (0.0%)	4 (0.0%)	21 (0.0%)	
7		2 (0.0%)	0 (0.0%)	2 (0.0%)	
8		3 (0.0%)	0 (0.0%)	3 (0.0%)	
9		0 (0.0%)	0 (0.0%)	0 (0.0%)	

Statistics: ^1^Pearson’s *χ*^2^ test; ^2^Wilcoxon test

Medical diagnoses in participating skiers and controls were identified in the Swedish National Patient Register, based on the International Statistical Classification of Diseases and Related Health Problems-10 classification (ICD 8,9,10-SE) provided from Swedish hospitals. The following diagnoses’ codes were investigated: 345 = epilepsy in ICD 8 and 9-SE, and G40 = epilepsy and the following subgroups in ICD 10-SE: G40.0–9. Subjects with epilepsy diagnosis were adjusted for diagnosis of alcohol over-consumption (non-specific code 303 alcohol intoxication/dependence syndrome), a possible triggering factor for provoked seizures. All analyses of the cohort data were approved and followed guidelines set by the Ethical Review Board in Uppsala, Sweden (Dnr 2010/305), before the study began.

### Study Design and Animal Description of Experimental Study

**Fig. 1:**
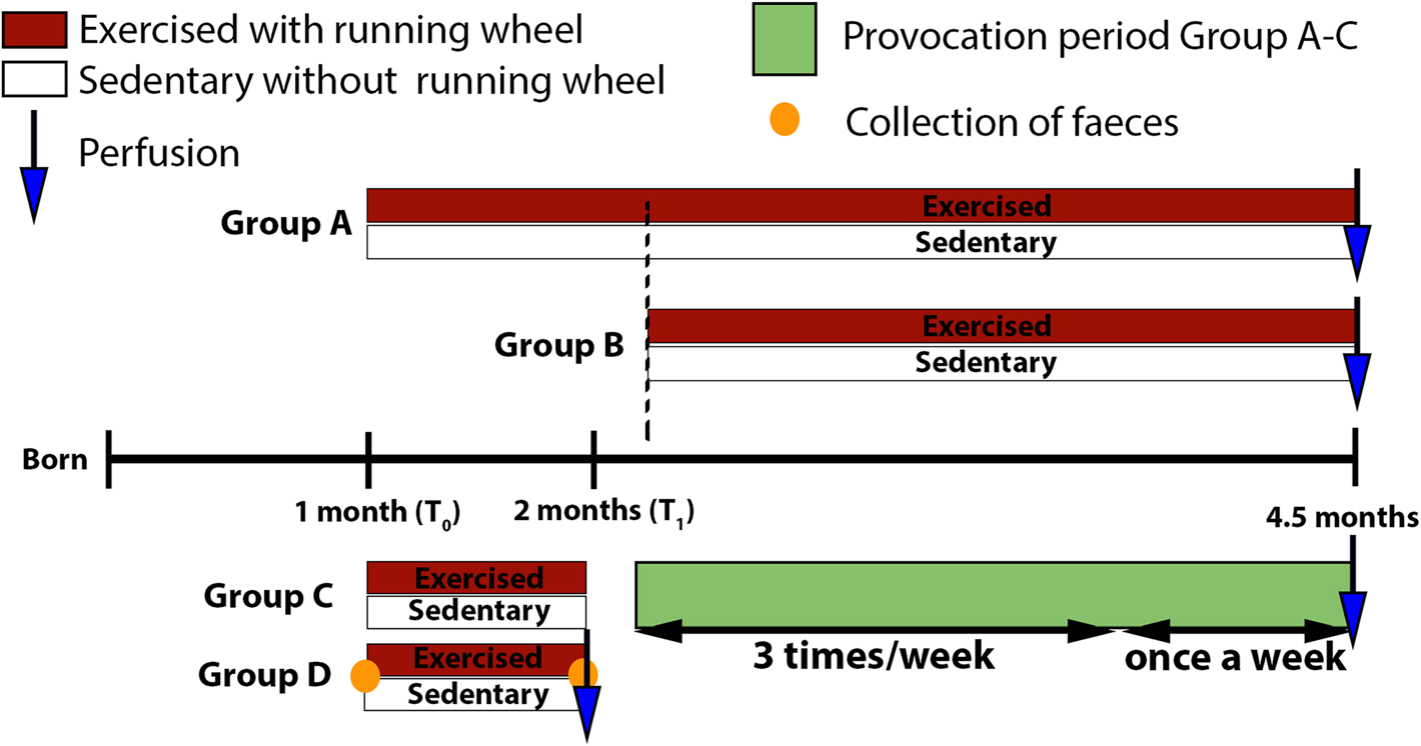
Animal assignment and study design. Group assignment (groups A–D) of the synapsin II knockout (SynIIKO) mice. Group A had running wheels in their home cage from 1 month of age until the end of experiment at 4.5 months of age, with 8 weeks of provocation starting at the age of 2.5 months. Group B had running wheels from 2.5 months of age (expected age of seizure onset) and throughout the 8-week provocation period. Group C had running wheels for 1 month starting at the age of 1 month, followed by 8 weeks of provocation without running wheels. Group D had running wheels for 1 month starting at the age of 1 month and were perfused at 2 months of age, before predicted seizure onset and received no provocations

The SynIIKO mice strain was developed by homologous recombination with ten generations of backcrossing to a C57/BL6 background strain [34, 35]. The study included males and females (in total *n* = 95), housed in same gender pairs with 12 h light/dark cycle and access to a standard pellet diet and water ad libitum*.* Both females and males were included in the experimental study to better match the clinical data and the epidemiology of epilepsy. This particular epilepsy model was chosen because of its well-defined time window of epileptogenesis and seizure development [34]. All animal procedures were approved and followed the guidelines set by the Local Ethical Committee at Lund University, Sweden (ethical number M93-14). Running wheels (diameter of 15.5 cm) were introduced in the standard housing cages (29 × 19 × 13 cm) and removed at different time points related to age and suspected seizure development; group A, running wheels 1–4.5 months of age; group B, running wheels 2.5–4.5 months of age; group C, running wheels 1–2 months of age; and group D, running wheels 1–2 months of age (Fig. 1; groups A–D). SynIIKOs without running wheels served as controls (sedentary). At around 2.5 months of age, SynIIKO mice develop handling-induced seizures [35]. As previously described [34], seizures were provoked by handling by the same 2 investigators, e.g. lifting the mouse once from one cage to another for maximum 10 s, between 2 and 4 pm, starting at 2.5 months of age in both exercised and sedentary mice (assigned to groups A, B or C). All handling were video-recorded and performed 1–3 times/week for an 8 week-provocation period in groups A, B and C. Group D was euthanized at 2 months of age before predicted seizure onset and received no provocations. The SynIIKO mice have a tendency to exhibit fewer seizures upon frequent provocations. In order to reduce the risk of seizure provocation during a postictal or refractory period and minimize the variability in seizure frequency among the different groups of mice, we decided to reduce the provocation frequency from 3 times a week after 5 weeks to once a week for the rest of the experiment. Seizures were quantified for all mice and analysed in terms of seizure frequency, severity and length over time.

#### Preparation and Analyses of Brain Tissue and Faeces From Mice

SynIIKO mice were deeply anesthetised with pentobarbital (200 mg/kg, i.p) and transcardially perfused with ice-cold saline (0.9%) followed by 4% paraformaldehyde (PFA). Brains were removed and stored in 4% PFA for 24 h before dehydration in 20% sucrose overnight and cut into 30 μm sections using a microtome (MICROM HM440E, Thermofisher). Brain sections were put in cryoprotective solution and stored at − 20 °C until use. Immunohistochemical stainings were performed for microglia (Iba1), astrocytes (GFAP) and neurons (Map2), newborn neurons (DCX), brain-derived neurotrophic factor (BDNF) and tyrosine receptor kinase B (TrkB) according to previous protocols [36]. The following primary antibodies were used: rabbit polyclonal anti-Iba1 (1:500 Wako, Japan), mouse monoclonal anti-GFAP (1:500 Bio-Rad, USA), rabbit polyclonal anti-Map2 (1:200, Santa Cruz, USA) and rabbit polyclonal anti-DCX (1:200, Abcam, UK), sheep polyclonal anti-BDNF (1:100 Santa Cruz, USA), mouse monoclonal anti-NeuN (1:500 Santa Cruz, USA) rabbit polyclonal anti-TrkB (1:100 Santa Cruz, USA), rabbit polyclonal anti-tubulin III (1:1000 Abcam, UK) and secondary antibodies; Alexa 488 goat anti-mouse, Cy3 goat anti-mouse, Cy3 goat anti rabbit, Cy3 donkey anti-sheep, biocytin goat anti-rabbit, Cy3-streptavidin (1:200, Jackson Laboratory, USA) Alexa 488 donkey anti-rabbit (1:200, Thermofisher, USA). Stained slices were coverslipped with nuclear stain Hoeschst (1:1000, Thermofisher) diluted in DABCO (Merck, Germany).

Mice faeces were collected between 8.00–10.00 AM before and 1 month after voluntary running in group D where mice had not yet developed seizures, to measure their corticosterone levels as a stress indicator. Protocol for corticosterone extraction from faeces was modified from Touma et al. [37]. Samples were dried at 37 °C overnight, grinded to powder, mixed with 80% methanol at 10% w/v for 30 min on a Vortex, and centrifuged at 2500×*g* for 15 min. Corticosterone levels were analysed by enzyme-linked immunosorbent assay (ELISA; Enzo Life Sciences, Solna, SE) according to manufacturer protocol.

Quantifications of the immunostainings were performed bilaterally in temporal lobe structures (hippocampus and entorhinal cortex (EC)), 3–4 brain sections/animal by researchers blinded to treatment conditions as previously described [34, 38, 39]. Number of Iba1+ microglial cells/sections in dentate gyrus, granule cell layer (GCL), and molecular layer (ML) of the hippocampus and EC were quantified manually due to low numbers of cells*.* Morphological analysis of microglia (ramified/surveying; small soma with several long processes, intermediate/activated; larger cell soma with fewer, thicker and retracting processes, and round/amoeboid/phagocytic; large soma with no processes) was performed in a subset of 120 Iba1^+^ cells/animal in dentate hilus, GCL and ML, separately, and 80 Iba1^+^ cells/animal in EC. GFAP of astrocytes, Map2 in neurons, BDNF, and TrkB expression were analysed by intensity measurements as the mean grey value (ImageJ software, NIH, USA). Numbers of Map2^+^ processes were manually counted in layer II of EC. Mean numbers of DCX^+^ newborn cells/section were quantified manually in the GCL and subgranular zone (SGZ) in the hippocampus.

### Statistics

Comparing the epidemiological datasets, a log-rank test with unadjusted hazard ratio (HR) from the Cox model was used and results presented as Kaplan-Meier curves or as confidence intervals (incidence in skiers and controls of diagnostic sub-codes for epilepsy). HR was later adjusted for previous alcohol diagnosis. Normal distribution of experimental data was analysed with Shapiro-Wilk test. Data with skewness was analysed with non-parametric Mann-Whitney test (Map2 and BDNF intensity). Immunohistochemical comparisons of 2 groups were performed using unpaired Student’s *t* test, except for corticosterone levels, where paired *t* test was used to match samples from the same animal. Seizure onset was analysed with a Fisher exact test. Iba1 morphology comparing 3 parameters was evaluated by two-way ANOVA with Bonferroni post hoc test. Experimental data was presented as absolute number, % or mean + SEM. All *p* values < 0.05 were considered statistically significant.

## Results

### Epidemiological Data

#### Reduced Incidence of Epilepsy in Physically Active Individuals After Participation in the Ski Race Vasaloppet

**Fig. 2:**
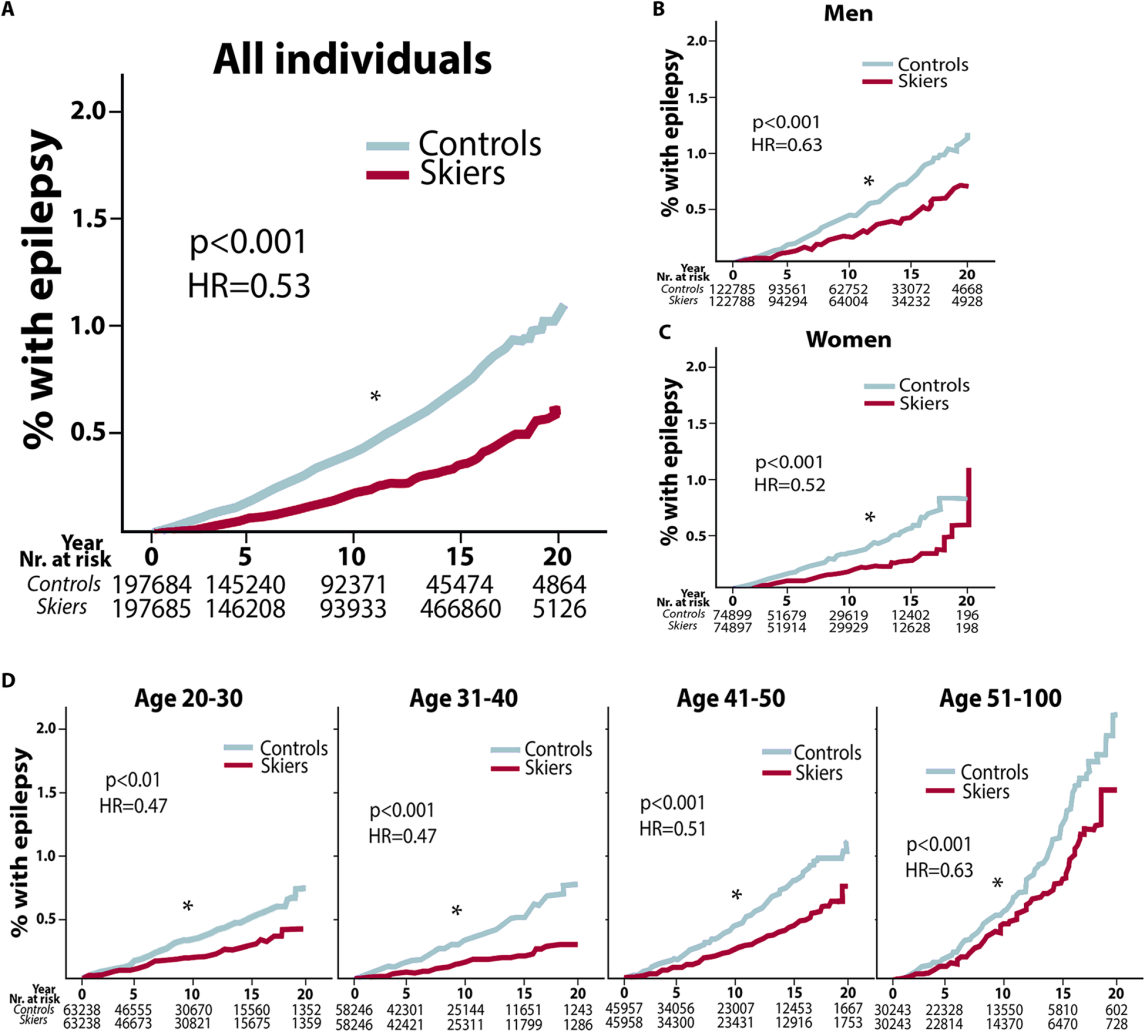
Epilepsy incidence divided according to gender and age groups. Data presented with Kaplan-Meier curves, with unadjusted hazard ration (HR). *Y*-axis represents incidence in %, and the *X*-axis presents follow-up time in years and number at risk. Overall incidence of epilepsy diagnoses at 0, 5, 10, 15 and 20 years after completed ski race in skiers compared to matched non-participating controls (**a**), in men (**b**) and women (**c**), in age groups 20–30, 31–40, 41–50 and 51–100 years (**d**). *p < 0.05

A cohort of 395,369 individuals was divided into 2 groups, participating skiers and non-participating controls. Both groups had equal numbers of men and women, age distribution and year of subject recruitment (Table 1 [29]). We found a significantly lower incidence of epilepsy (epilepsy diagnosis codes 345 or G40) among skiers following up to 20 years after their participation in the ski race compared to non-participating controls (*n* = 424 in the skiing group compared to *n* = 789 in control group, *p* > 0.001). Since none of the individuals were diagnosed with epilepsy before entering the study, the data imply a reduced incidence of epilepsy of almost 50% at 20 years (Fig. 2a).

#### Both Men and Women, at All Ages, Exhibited Reduced Incidence of Epilepsy After Participation in Vasaloppet

Reduction in epilepsy incidence was present in both men (Fig. 2b) and women (Fig. 2c), with almost 40% reduction in men and 50% reduction in women over a 20-year time period. When dividing the participants into age groups (20–30, 31–40, 41–50 and 50–100 years), 40–50% decreased incidence remained in all age groups (Fig. 2d, bottom row).

#### Faster Skiers Participating in Vasaloppet Revealed Lower Incidence of Epilepsy Compared to Slower Skiers

**Fig. 3:**
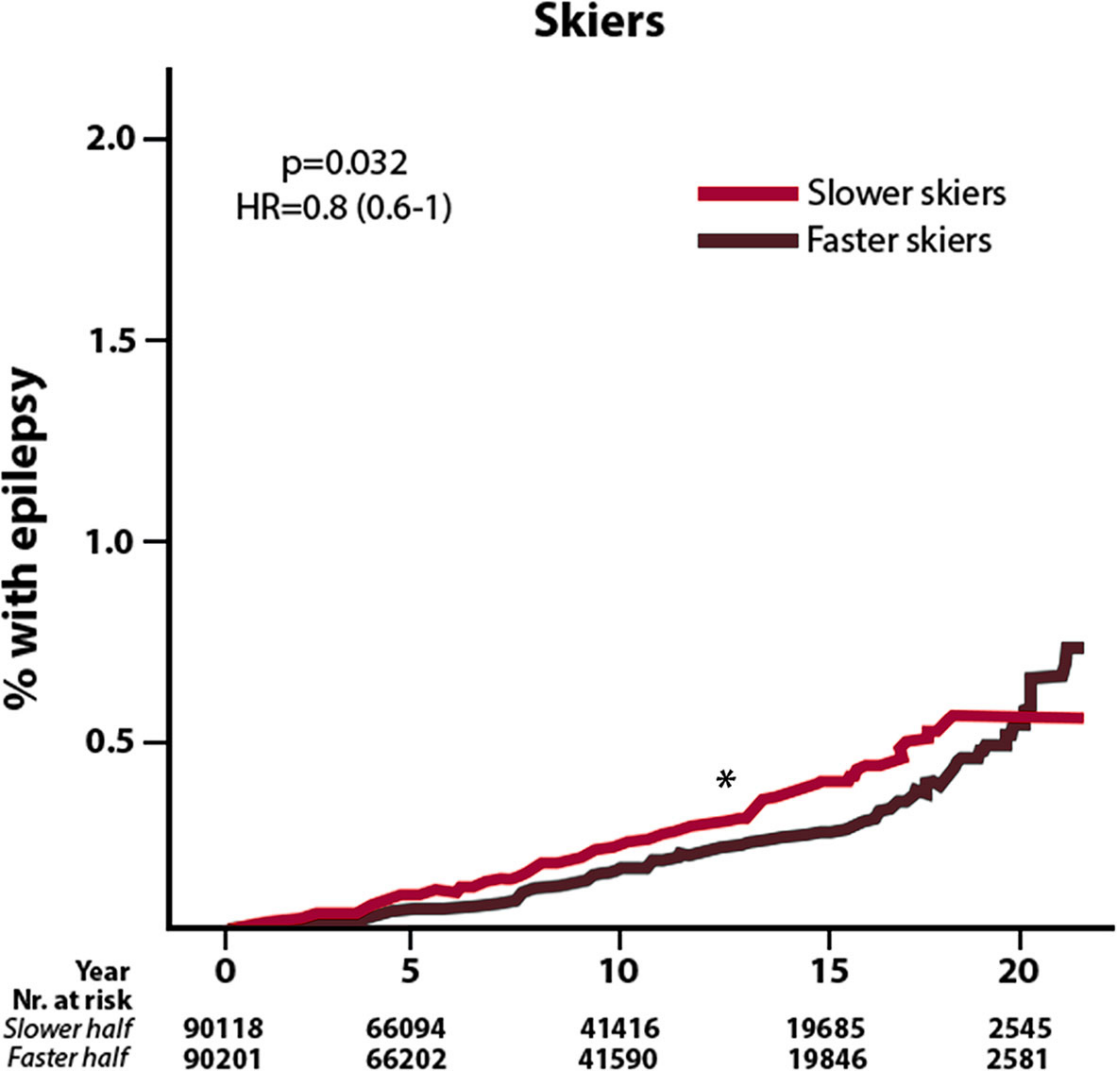
Epilepsy incidence in faster compared to slower skiers. Data presented with Kaplan-Meier curves, with unadjusted hazard ration (HR). *Y*-axis represents incidence in %, and the *X*-axis presents follow-up time in. Skiers where divided into slower skiers, with a finishing time below the median and faster skiers, with a finishing time above the median. *p < 0.05

The epilepsy incidence was further reduced among faster skiers, defined as skiers with a race time below the median, compared to slower skiers with a race time above the median (Fig. 3). The actual reduction between faster and slower skiers was relatively small (20%).

#### Partial and Unspecified Epilepsy Diagnoses were Reduced in Skiers Participating in Vasaloppet

**Fig. 4:**
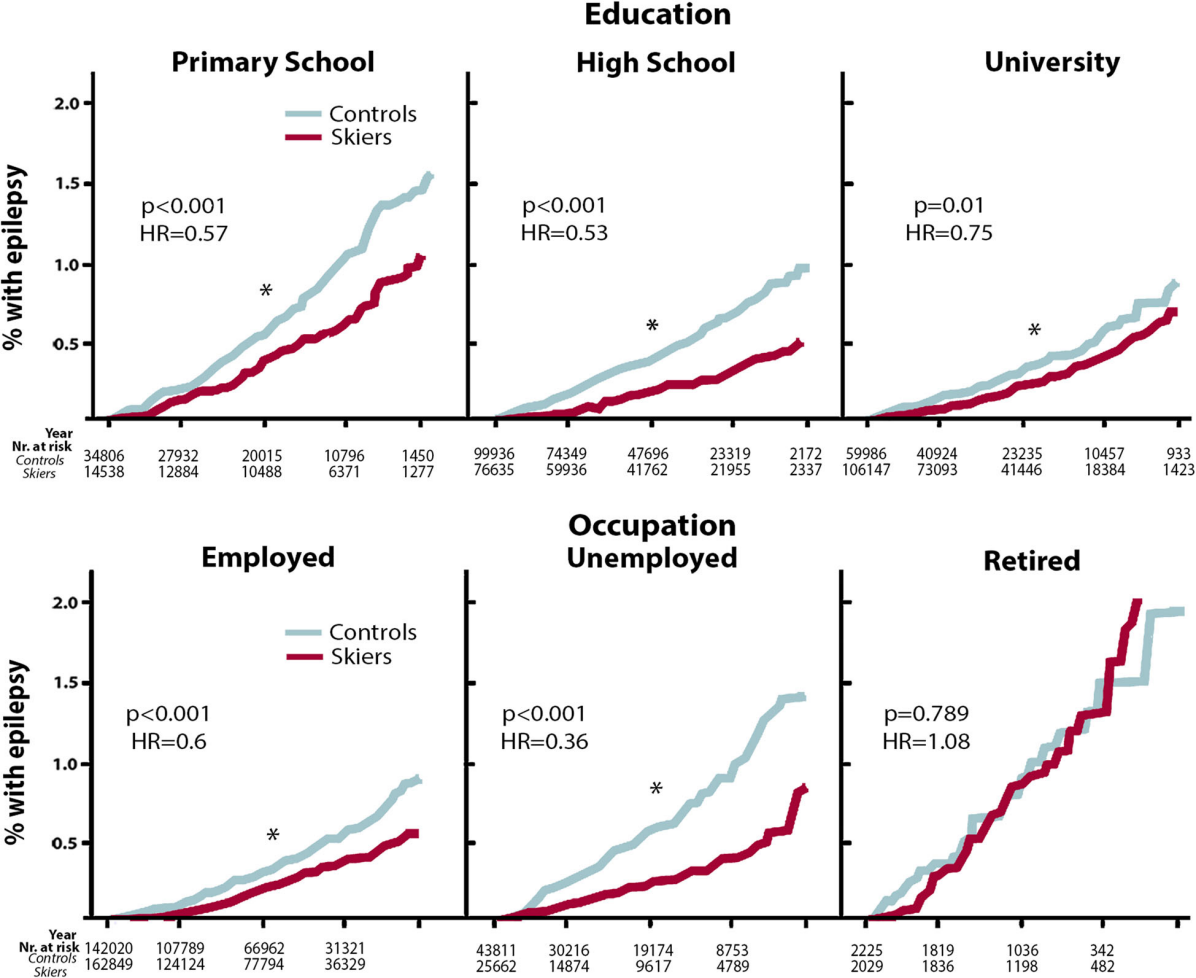
Epilepsy incidence divided according to educational or occupational level. Data presented with Kaplan-Meier curves, with unadjusted hazard ration (HR). *Y*-axis represents incidence in %, and the *X*-axis presents follow-up time in years and number at risk. Incidence of epilepsy diagnoses at 0, 5, 10, 15 and 20 years after completed ski race in skiers compared to matched non-participating controls with primary school, high school and university as highest education level (**a**, upper row) and according to occupation level (employed, unemployed, retired) (**b**, lower row). *p < 0.05

We divided the epilepsy patients according to epilepsy sub-codes (ICD-10 G40.1-9). Subgrouping reduced n-values, but a significant reduction was still present for the G40.2 diagnosis code of partial symptomatic epilepsy and epileptic syndromes with complex partial seizures and the largest group of G40.9 (unspecified epilepsy) in skiers compared to controls (Table 2). In order to reduce a confounding effect of alcohol-associated seizures, subjects with current/previous alcohol diagnosis were excluded from G40.9 group, but still the reduction in epilepsy incidence remained (CI: 0.21–0.27 controls vs 0.1–0.14 skiers). Even if skiers had higher education and rate of employment compared to controls (Table 1), the reduced epilepsy incidence remained when subdividing individuals according to education and occupational level (Fig. 4). The single subgroup lacking differences in epilepsy incidence was the relatively small group of retired subjects (1.2% controls and 1.1% skiers, Fig. 4, lower right).

**Table 2:** Epilepsy incidence in subgroups of epilepsy diagnoses. Incidence rate/1000 person years and confidence intervals after unadjusted log rank test on subgroups of individuals (> 25 events per diagnosis) with ICD-10 diagnosis codes G40.0-9 with unadjusted HR and with HR adjusted for current or previous alcohol diagnosis. *Significant differences

Rate. tab	*N* at risk	*N*, event	Persons years	Inc. rate	Conf. int
Epilepsy, G40.1: partial symptomatic epilepsy and epileptic syndromes with simple partial seizures					
Controls	140,919	26	1,003,770	0.03	(0.02, 0.04)
Skiers	140,920	21	1,009,001	0.02	(0.02, 0.03)
Epilepsy, G40.2: partial symptomatic epilepsy and epileptic syndromes with complex partial seizures					
Controls	140,919	55	1,003,621	0.05	(0.04, 0.07)
Skiers	140,920	34	1,008,946	0.03	(0.02, 0.05)*
Epilepsy, G40.3: Generalized idiopathic epilepsy and epileptic syndromes					
Controls	140,919	16	1,003,807	0.02	(0.01, 0.03)
Skiers	140,920	12	1,009,001	0.01	(0.01, 0.02)
Epilepsy, G40.9: unspecified epilepsy					
Controls	140,919	263	1,002,884	0.26	(0.23, 0.3)
Skiers	140,920	125	1,008,638	0.12	(0.1, 0.15)*
Epilepsy G40.9 excl: alcohol diagnosis					
Controls	140,044	233	991,845	0.23	(0.21, 0.27)
Skiers	140,574	121	1,005,032	0.12	(0.1, 0.14)*

### Experimental Data

#### Reduced Seizure Frequency and Delayed Seizure Onset in Synapsin II Knockout Mice Following Early Voluntary Running

**Fig. 5:**
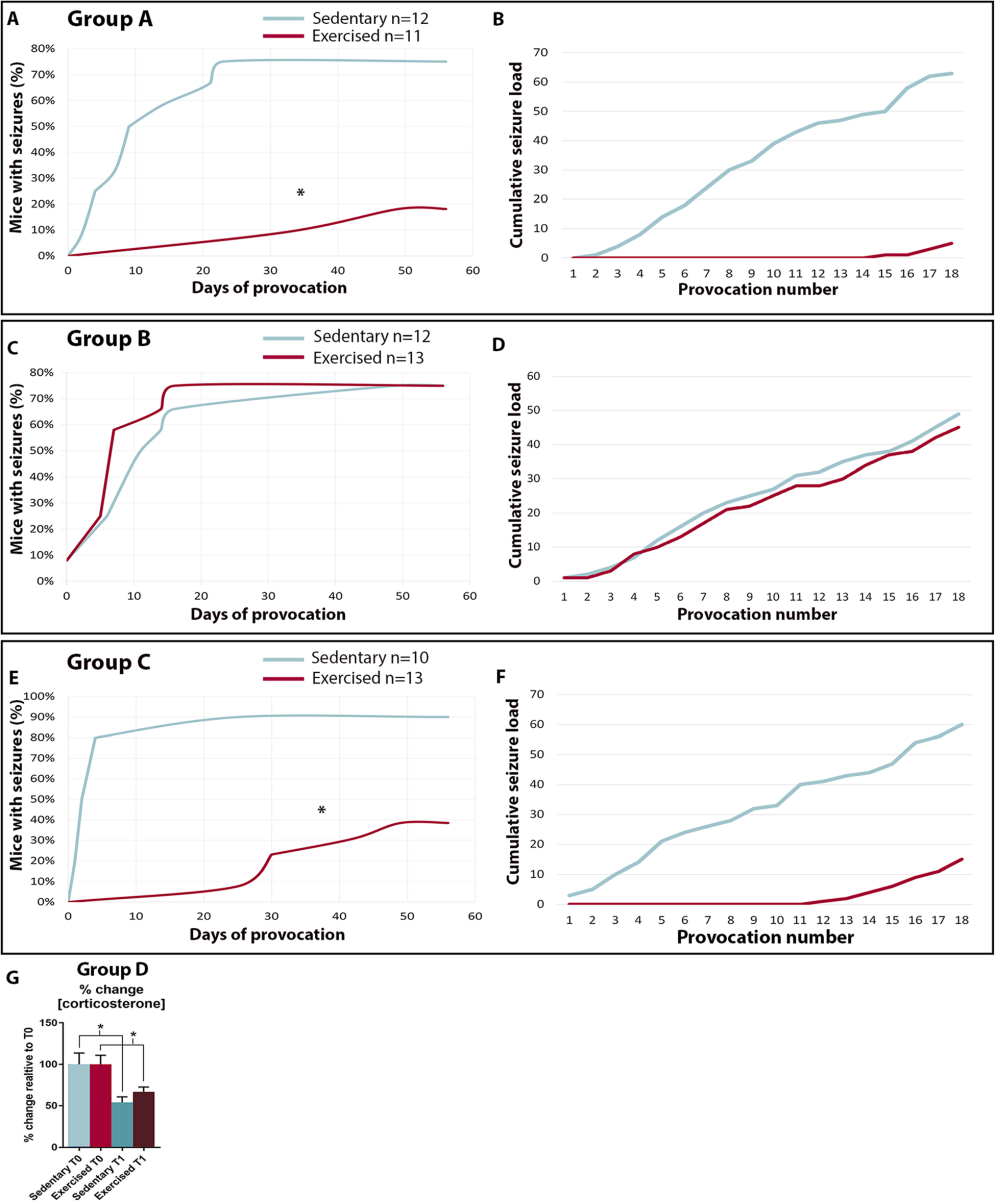
Delayed seizure onset in synapsin II knockout (SynIIKO) mice following voluntary running. Percentage of SynIIKO mice with or without voluntary running (exercised and sedentary group) exhibiting provoked seizures (**a**, **c**, **e**) and cumulative seizure load during the provocation period (**b**, **d**, **f**). Group A starting 1.5 months before the 8-week long provocation period (**a**, **b**). Group B voluntary running for 2 months starting concurrently with seizure provocations (**c**, **d**). Group C voluntary running for in total 1 month starting 1 month before provocation period (**e**, **f**). Corticosterone level in both the sedentary and exercised group was reduced at 2 months compared to 1 month of age. However, no differences were observed in corticosterone levels in faeces before (T_0; mice 1 month of age_) and after (T_1; mice 2 months of age_) 1 month of voluntary running between sedentary and exercised mice (**g**). *p < 0.05, **a**–**c** Fisher exact test and group D paired Student’s *t* test. Group A *n*_sedentary_ = 12 and *n*_exercised_ = 11, group B *n*_sedentary_ = 12 and *n*_exercised_ = 13, group C *n*_sedentary_ = 10 and *n*_exercised_ = 13, group D *n*_sedentary_ = 12 and *n*_exercised_ = 12

Placement of a running disc in the home cages of SynIIKO mice resulted in almost instantaneous excessive running of all mice, noticed by daily visual observations. Voluntary running starting at the age of 1 month, hence before the expected seizure onset (at 2.5 months), and continuing until 4.5 months of age significantly delayed seizure onset in the SynIIKO mice (Fig. 5a, group A; *p* = 0.04). The majority of exercising SynIIKO mice in group A did not develop seizures during the provocation period (with seizures: 3 out of 11 exercised compared to 9 out of 12 animal sedentary groups). Running wheels introduced later, at the expected seizure onset (at 2.5 months of age) and continuously present during the provocation period, had no effect on seizure development or frequency (Fig. 5c, group B; *p* > 0.99). In group C, the SynIIKO mice received access to running wheels for a period of only 1.5 months before expected seizure onset (at 2.5 months) followed by no access during the 8-week provocation period (Fig. 5e, group C; *p* = 0.01). During the first 4 weeks of provocations, the percentage of mice in group C that developed seizures in the exercised group was again less than 20% (with seizures: 3 out of 13 exercised compared to 8 out of 10 sedentary). The percentage started to rise during the last 4 weeks of provocations, but remained at 40% at the end of the experiment (with seizures: 5 out of 13 exercised, compared to 9 out of 10 in the sedentary SynIIKO mice). Cumulative seizure load during the provocation period for exercised and sedentary groups is presented in Fig. 5b, d and f. Total seizure load did not differ between males and females in sedentary and exercised groups A, B and C, respectively (group A: sedentary males 5.6 ± 1.4 vs sedentary females 4.5 ± 2.1, exercised males 1.0 ± 1.4 vs exercised females 0.0 ± 0.0; group B: sedentary males 6.5 ± 0.5 vs sedentary females 4.3 ± 1.9, exercised males 1.9 ± 2.1 vs exercised females 0.17 ± 1.17; group C: sedentary males 5.2 ± 1.2 vs sedentary females 1.8 ± 1.2, exercised males 2.2 ± 1.0 vs exercised females 5.3 ± 1.3).

#### Consistent Seizure Semiology in Synapsin II Knockout Mice Following Voluntary Running

All SynIIKO mice exhibited similar stereotypic seizure semiology, hence no differences in seizure severity could be observed. The seizures started with facial/ear twitching and chewing for 5–10 s (grades 0–2 on the Racine scale [40]), followed by tonic clonic movements of trunk and limbs (grade 5 on Racine scale) for about 10 s. In the end of the seizure, animals reverted to focal symptoms such as chewing, drawling and vocalization (grades 0–2 in the Racine scale) for 30–40 s. The average seizure length did not differ between exercised and sedentary mice, and they all experienced the same semiology as described above. In group A, the exercised SynIIKO mice had few seizures for statistical analyses of seizure length. In groups B and C, no differences in seizure length were observed (group B, sedentary 57 ± 6 vs exercised 49 ± 6 s; group C, sedentary 60 ± 7 vs exercised 52 ± 5 s).

#### Early Voluntary Running did not Change Corticosterone Levels in Faeces of Synapsin II Knockout Mice

As a stress level readout [41], faeces were collected from SynIIKO mice at 1 and 2 months of age, before seizure onset, after 1 month of voluntary running. Potential confounding effects induced by seizures were thereby avoided. Measurements of corticosterone levels in morning faeces, showed no differences between exercised and sedentary mice (Fig. 5g, group D).

#### No Alterations In Microglial and Astroglial Cell Activation in Hippocampus and Entorhinal Cortex Of Synapsin II Knockout Mice Following Early Voluntary Running

**Fig. 6:**
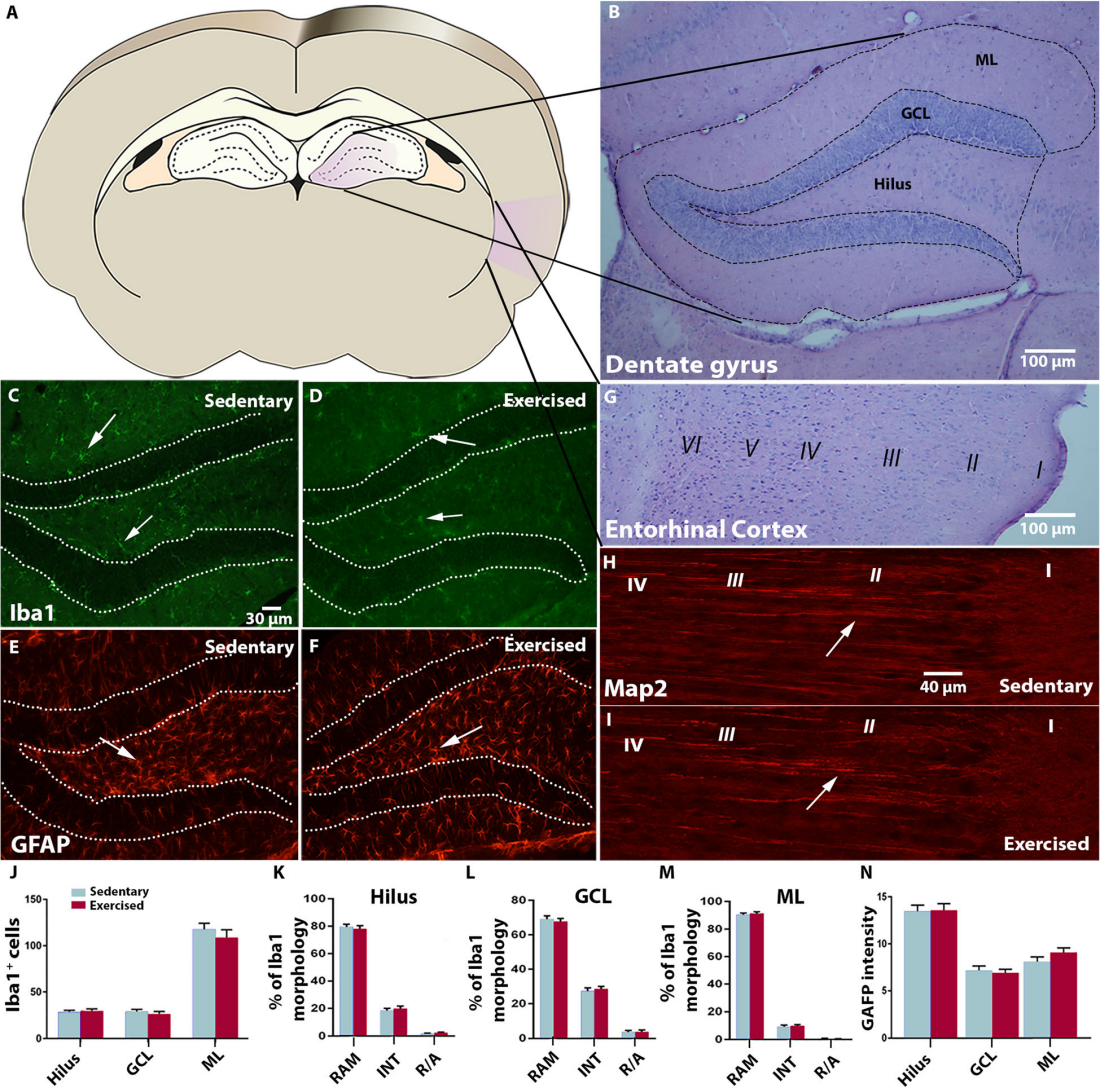
Microglia number and morphology, astrocytic GFAP expression and neuronal Map2 intensity in SynIIKO mice after 1 month of voluntary running before seizure onset. All stainings were evaluated in coronal brain sections, and areas included in the analyses are highlighted in purple (**a**). Photomicrograph of a haematoxylin/eosin staining shows gross histology of the dentate gyrus in the hippocampus of SynIIKO mice at the age of 2 months (group D) (**b**). Representative immunohistochemical images of Iba1^+^ and GFAP^+^ cell distribution (white arrows) in sedentary (**c**, **e**) and exercised (**d**, **f**) SynII KO mice, respectively. Haematoxylin/eosin staining of entorhinal cortex with the six cortical layers indicated in roman numbers (**g**). Map2 immunohistochemical staining of entorhinal cortex in sedentary (**h**) and exercised (**i**) SynIIKO mice. Representative Map2^+^ neuronal fibres in entorhinal cortical layer II are indicated by arrows (**h-i**). Numbers of Iba1^+^ microglial cells in the dentate hilus, granule cell layer (GCL) and molecular layer (ML) of the hippocampus in sedentary and exercised SynIIKO mice (**j**). Percentage of ramified (RAM), intermediate (INTER) and amoeboid/round (A/R) morphologies of Iba1^+^ cells in the hilus (**k**), GCL (granule cell layer (**l**)) and ML (molecular layer (**m**)). Intensity measurements of GFAP immunohistochemical staining in astrocytic processes within the hilus, GCL and ML of the hippocampus (**n**)

We have previously observed changes related to microglial activation in SynIIKO mice compared to wildtype mice both before and after seizure development [34]. Since seizures per se can lead to pathophysiological changes including neuroinflammatory reactions [42], we decided to evaluate the histological effects of voluntary running in the SynIIKO mice at 2 months of age, prior to seizure development, e.g. reducing the confounding effect related to number of seizures. Iba1^+^ microglial cells were examined in sub-regions of the dentate gyrus in the hippocampus (Fig. 6a–d) and EC (Fig. 6g) in SynIIKO mice with or without a prior month of voluntary running. No differences in numbers or morphology of Iba1^+^ cells were observed between exercised and sedentary SynIIKO mice in either hippocampus (Fig. 6j) or EC (124 ± 5 in sedentary vs. 114 ± 10 cells/brain section in exercised group) (Fig. 6k–m). Astroglial activity measured by GFAP intensity in the dentate gyrus (Fig. 6n) and EC (mean grey value 14.0 ± 0.7 sedentary vs. 13.7 ± 0.3 exercised group) was not affected and neuronal dendrite intensity measured by Map2 intensity in the dentate gyrus (mean grey value in hilus; 23.4 ± 0.9 sedentary vs 23.4 ± 0.8, GCL; 18.5 ± 0.9 sedentary vs 20.1 ± 0.7 exercised, ML; 42.4 ± 0.7 sedentary vs 41.8 ± 1.1 exercised) and numbers of Map2^+^ processes in EC (50 ± 1 sedentary vs. 44 ± 4 exercised) remained unaltered (Fig. 6h, i).

#### Increased Number of Newborn Neurons Within the Hippocampus Following 1 Month of Voluntary Running Before Seizure Onset in Synapsin II Knockout Mice

**Fig. 7:**
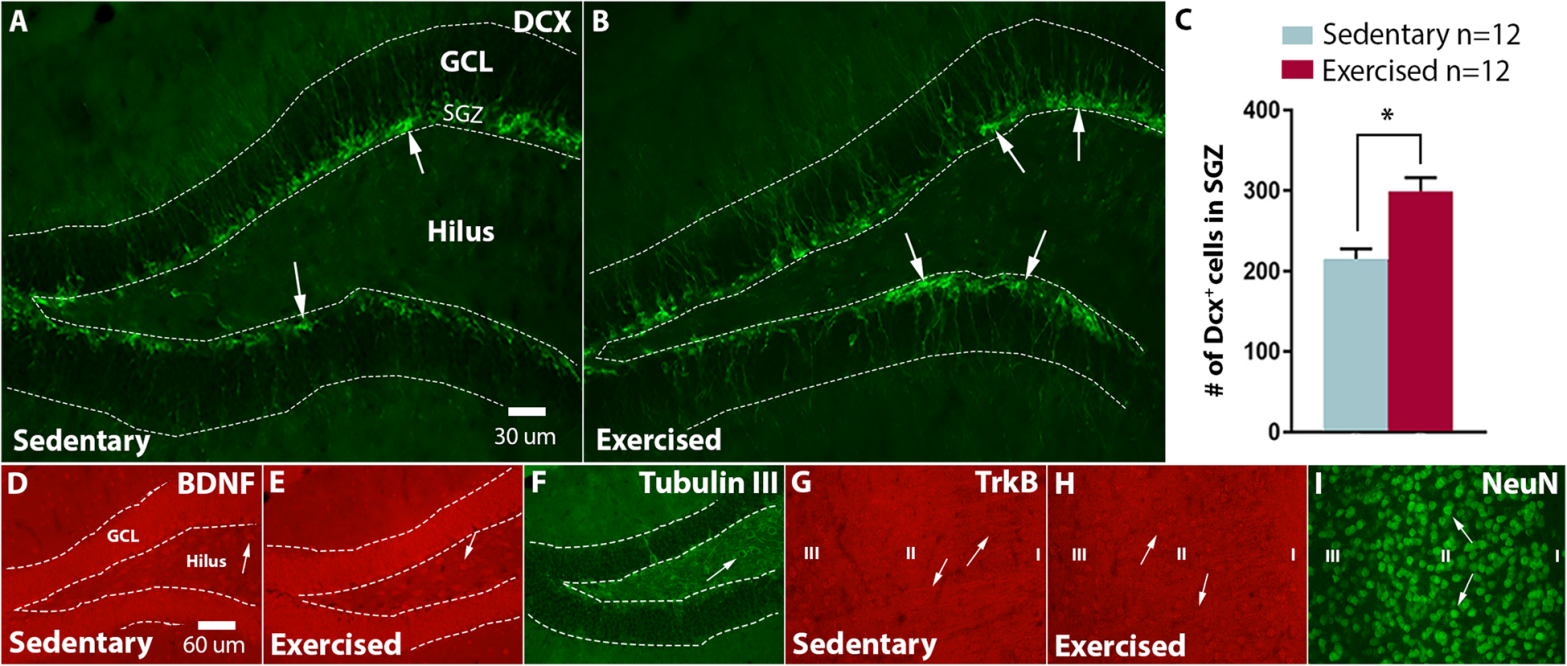
Numbers of newborn neurons in the hippocampus and BDNF/TrkB expression in hippocampus and entorhinal cortex of SynIIKO mice after 1 month of voluntary running before seizure onset. Distribution of DCX^+^ cells within the subgranular zone (SGZ) of the dentate gyrus in the hippocampus in exercised mice as compared to sedentary SynIIKO mice (group D) (**a**, **b**). Number of DCX^+^ cells as mean number of cells/brain section (**c**). BDNF expression in the dentate hilus, GCL and ML of the hippocampus in sedentary and exercised SynIIKO mice (**d**, **e**), with representative region of interest delineated also in tubulin III (**f**). TrkB expression in the entorhinal cortex in sedentary and exercised SynIIKO mice (**g**, **h**), with representative region of interest indicated also in NeuN staining (**i**). *p < 0.05, unpaired Student’s *t* test, *n*_sedentary_ = 12 and *n*_exercised_ = 12

SynIIKO mice have reduced hippocampal neuroblast production at 2 months of age compared to wild types [34]. Following 1 month of voluntary running prior to predicted seizure onset, numbers of DCX cells within the sub-granular zone of the dentate gyrus increased by 40% compared to sedentary controls (Fig. 7a–c). BNDF and its high affinity receptor TrkB have shown significant contribution to neurogenesis [43]. However, when investigating the BDNF and TrkB intensity in the hippocampus and EC, no differences were observed (Fig. 7d–i). Mean grey value BDNF in hilus: 47.4 ± 2.5 sedentary vs 42.2 ± 2.5 exercised, GCL: 53.6 ± 2.3 sedentary vs 50.6 ± 1.8 exercised, ML: 44.8 ± 2.1 sedentary vs 41.0 ± 2.1 exercised, EC: 49.6 ± 2.4 sedentary vs 45.5 ± 2.2 exercised. Mean grey value TrkB in hilus: 21.4 ± 1.7 sedentary vs 18.0 ± 3.0, exercised, GCL 21.5 ± 1.5 sedentary vs 19.1 ± 2.8 exercised, ML 24.6 ± 1.6 sedentary vs 20.9 ± 0.95 exercised, EC 30.0 ± 1.8 sedentary vs 26.7 ± 1.2 exercised.

## Discussion

We analysed a cohort of 197,685 skiers, participating in one of the largest cross-country ski races in the world, Vasaloppet, and compared their epilepsy incidence to 197,684 non-participating controls from the general Swedish population. Patients with epilepsy, severe diseases such as stroke and chronic neurological diseases, or previous/present alcohol diagnosis before entering the race were excluded. We observed a robust decrease in the incidence of epilepsy, from > 1% to < 0.6%, during a follow-up period of up to 20 years. The effect was observed in both genders, at all ages until retirement, including all education levels and regardless of employ/unemployment status. The vast majority of individuals exhibited the unspecified diagnosis of epilepsy, G.40.9, which contains various numbers of epilepsies. However, even when dividing the cohort into smaller subgroups of specific epilepsy diagnosis, the reduced epilepsy incidence remained in partial and unspecified epilepsy. Skiers with faster compared to slower finishing times in the race displayed lower incidence of epilepsy.

We also explored the effect of physical activity on the development of epilepsy in a genetic epilepsy mouse model of presumably focal epilepsy with secondary generalization, with the following conclusions: (i) seizure development was reduced 5-fold in SynIIKO mice following 3.5 months of voluntary running, (ii) the reduced development of seizures was dependent on early voluntary running for 1 month before predicted seizure onset, (iii) voluntary running for 2 months had no anti-convulsive effect once seizures had developed and (iiii) previously defined brain pathologies in SynIIKO mice such as microglial and astrocytic activation was not altered by early running, while hippocampal neurogenesis was increased without an increase in BDNF or TrkB expression.

The majority of Vasaloppet participants were at the time of the race assumed to be in good physical condition, which is associated with an overall healthy lifestyle, including exercising regularly [33], confirmed in a digital survey of the participants in 2006 [33]. In agreement with our present findings, a retrospective study including 1.2 million individuals from basic Swedish military enrolment from year 1968–2005, concluded that men with a high vascular fitness at the age of 18 had lower incidence of epilepsy compared to subjects with low vascular fitness 37 years later (HR 1.39 vs 1.65, diagnose codes 345 in ICD-8,9 and G40–41 in ICD-10 [44]). The Vasaloppet cohort gives a unique opportunity to study the incidence of diseases in physically active skiers compared to the common population of Sweden. We have previously shown that this cohort has a lower incidence of death from all causes [28], a decreased risk of recurrent myocardial infarction, while the risk for recurrent stroke was similar to that of non-participants [29, 30]. Even incidence of cancer epidemiologically associated with life style factors including smoking, body weight, dietary habits, and physical activity was decreased [45]. Similar to our previous studies [28, 29], the incidence of epilepsy, was reduced in individuals with the faster compared to slower finishing times, indicating an association between degree of aerobic fitness and epilepsy incidence.

Our study has limitations related to retrospective registry studies. Missing or unavailable data, such as continuous lifestyle information and medical diagnosis from out-patient care, cannot be accounted for here. Furthermore, the participating skiers were assumed to be healthier than the non-participating controls, according to a survey in 2007 [33]. However, the survey compared information from the skiers to the Swedish Health register and not specifically to the matched control group. Hence, we cannot confirm a significant difference within the cohort. The life style confounders that were accounted for were (1) family status, (2) education level, and (3) occupation level, and (4) partly patients with alcohol-related diagnostic codes.

In spite of the heterogeneous nature of epilepsy, a pre-selection of possible epilepsy diagnoses in the cohort was likely. Many genetic epilepsies, epilepsies caused by extensive brain malformations and early-onset epilepsies have manifested with/without neurological deficits already before the age of 18 and, hence, excluded in the current study. The reduced incidence of focal and unspecified epilepsy in the ski race participants (age 20–100 years) points towards an effect on acquired forms of epilepsy. The magnitude of the reduced epilepsy incidence in the skiers is one of the largest differences measured among the medical diagnosis investigated in the entire cohort of participants. As such, it cannot only be explained by reduced incidence of the most common predisposing disorders such as stroke [30] or head trauma. The relatively long follow-up time of 20 years makes the results even more robust.

The lack of a protective anti-convulsive effect of voluntary running in the SynIIKO mice was unexpected. Previous studies with chemically induced models of more severe convulsive status epilepticus have suggested an anti-convulsive effect with reduced seizure frequency following 1–2 months of exercise starting 3 days–4 months after the initial spontaneous recurrent seizure [9, 19, 46, 47]. The inconsistent results may be related to differences in origin and severity of the experimental epilepsy models. In contrast to chemically/electrically induced acquired convulsive seizure models, the stress-associated convulsive seizures in SynIIKO mice arise as a result of predisposing features associated with the genetic mutation. Even if 1 month of voluntary running before predicted seizure onset in the SynIIKO mice was enough to delay the occurrence of seizures, it did not completely hinder seizure development in the long term. Epileptogenesis in the SynIIKO mice was further diminished when voluntary running was applied both before and throughout the provocation period, which suggests that the beneficial effect of a physically active life style is related to predisposing epileptogenic features of epilepsy. In support, a previous study on rats subjected to a complex enriched environment including free access to a running wheel, demonstrated a delayed kindling effect upon electrical stimulation when the environment was introduced before—but not after—stimulation procedures had started [48].

The previously described immune reaction in the brain of SynIIKO mice [34] was not altered following 1 month of voluntary running, which suggests that either more specific immune signalling pathways are involved or other predisposing features are more important for the exercise-induced beneficial effect on seizure onset. Graban and colleagues have reported increased expression of glutamate transporters in cortex of voluntary exercising rats [49], which could lead to a more effective clearance of excessive glutamate in the synapse. Since docking of glutamate-containing synaptic vesicles is impaired in SynIIKO mice [50], the effect on synaptic glutamate levels following physical activity may counteract the excitatory-inhibitory imbalance seen in SynIIKO mice. Apart from seizure development, SynIIKO mice also manifest autistic-related behaviour and, with age, cognitive decline [51, 52]. Possible cognitive and psychiatric effects related to the synaptic changes are interesting topics for future studies.

There is strong previous evidence for a running-induced increase in neurogenesis in mice [53–55] as well as increase in neurotrophic responses [56]. In this study, both BDNF and TrkB were unaltered but the regenerative response from a physical active life style counteracted a decreased neuronal production that precedes seizure onset in SynIIKO mice. The production of new neurons in SynIIKO mice increases again after seizure onset [34] and seizure-induced neurogenesis is also well described in several other seizure models [57, 58]. Whether the exercise-induced altered neurogenesis during the epileptogenic phase before seizure onset reflects or is associated with the differences in seizure susceptibility remains to be clarified.

## Conclusion

Here, we describe reduced incidence of epilepsy in an adult human population of physically active skiers. The experimental data suggests that early voluntary physical activity before predicted seizure onset may inhibit or delay epilepsy development in a genetic mouse model of epilepsy.

## Data Availability

All animal data used or analysed during the current study are available from the corresponding author on reasonable request. The cohort dataset analysed in this study is not publicly available due to the integrity of the participants, but are available from the corresponding author on reasonable request.

## Abbreviations

ARRIVE: Animal Research: Reporting of In Vivo Experiments
BDNF: Brain-derived neurotrophic factor
CA2,-3: *Cornu Ammonis* 2,-3
CI: Confidence interval
DCX: Doublecortin
EC: Entorhinal cortex
ELISA: Enzyme-linked immunosorbent assay
GCL: Granule cell layer
GFAP: Glial fibrillary acidic protein
HPA: Hypothalamic-pituitary-adrenal
HR: Hazard ratio
Iba1: Ionized calcium binding adaptor molecule 1
IDC-10: International Statistical Classification of Diseases and Related Health Problems-10 classification
Map2: Microtubule-associated protein 2
ML: Molecular layer
PFA: Paraformaldehyde
SEM: Standard error of mean
Syn II KO: Synapsin II knock out
TrkB: Tyrosine receptor kinase B
